# Tick-Borne Encephalitis Virus Infection Alters the Sialome of *Ixodes ricinus* Ticks During the Earliest Stages of Feeding

**DOI:** 10.3389/fcimb.2020.00041

**Published:** 2020-02-18

**Authors:** Charles E. Hart, Jose M. Ribeiro, Maria Kazimirova, Saravanan Thangamani

**Affiliations:** ^1^SUNY Center for Environmental Health and Medicine, SUNY Upstate Medical University, Syracuse, NY, United States; ^2^Institute for Global Health and Translational Science, SUNY Upstate Medical University, Syracuse, NY, United States; ^3^The Institute for Translational Science, University of Texas Medical Branch, Galveston, TX, United States; ^4^Laboratory of Malaria and Vector Research, National Institute of Allergy and Infectious Diseases, Bethesda, MD, United States; ^5^Institute of Zoology, Slovak Academy of Sciences, Bratislava, Slovakia; ^6^Department of Microbiology and Immunology, SUNY Upstate Medical University, Syracuse, NY, United States

**Keywords:** vector-skin interface, tick-borne encephalitis virus, tick sialome, tick feeding, immunomodulation

## Abstract

Ticks are hematophagous arthropods that transmit a number of pathogens while feeding. Among these is tick-borne encephalitis virus (TBEV), a flavivirus transmitted by *Ixodes ricinus* ticks in the temperate zone of Europe. The infection results in febrile illness progressing to encephalitis and meningitis with a possibility of fatality or long-term neurological sequelae. The composition of tick saliva plays an essential role in the initial virus transmission during tick feeding. Ticks secrete a diverse range of salivary proteins to modulate the host response, such as lipocalins to control the itch and inflammatory response, and both proteases and protease inhibitors to prevent blood coagulation. Here, the effect of viral infection of adult females of *Ixodes ricinus* was studied with the goal of determining how the virus alters the tick sialome to modulate host tissue response at the site of infection. Uninfected ticks or those infected with TBEV were fed on mice and removed and dissected one- and 3-h post-attachment. RNA from the salivary glands of these ticks, as well as from unfed ticks, was extracted and subjected to next-generation sequencing to determine the expression of key secreted proteins at each timepoint. Genes showing statistically significant up- or down-regulation between infected and control ticks were selected and compared to published literature to ascertain their function. From this, the effect of tick viral infection on the modulation of the tick-host interface was determined. Infected ticks were found to differentially express a number of uncategorized genes, proteases, Kunitz-type serine protease inhibitors, cytotoxins, and lipocalins at different timepoints. These virus-induced changes to the tick sialome may play a significant role in facilitating virus transmission during the early stages of tick feeding.

## Introduction

Ticks are obligate hematophagous arthropods of the order Ixodida that transmit a variety of disease agents to humans and animals. In Europe, Russia, and Northern Asia, tick-borne viral diseases are a major public health concern. Of particular concern is tick-borne encephalitis virus (TBEV), a flavivirus endemic to Europe and northern Asia which is vectored by *Ixodes ricinus* in Europe and *Ixodes persulcatus* in parts of Eastern Europe and Asiatic Russia. Upon TBEV-infected tick feeding, the virus initially replicates in host skin fibroblasts (Hermance et al., 2016) and macrophages (Labuda et al., 1996). The virus is then transported by these macrophages to the draining lymph nodes, where it replicates before entering the blood and progressing to the remainder of the body. This results in febrile illness that may progress to neuroinvasive disease with meningitis or in more severe cases to encephalitis or myeloencephalitis (Zavadska et al., 2018). Tick-borne encephalitis (TBE) is diagnosed in about 12,000 people per year (World Health Organization, 2011), although the rate of exposure is thought to be substantially higher. The fatality rate of the European subtype of TBE (transmitted by *I. ricinus*) is 1–3%, with over 40% of neuroinvasive cases resulting in long-term neurological sequelae.

Ticks are pool-feeders. To facilitate the feeding process, ticks secrete to the feeding pool a complex mixture of compounds in their saliva, containing hundreds of proteinaceous, and non-proteinaceous molecules that modulate host hemostasis, inflammation, immune reactions, and wound healing. The expression of the tick salivary factors undergoes significant and dynamic temporal regulation, where different factors are expressed and secreted throughout the feeding process (Francischetti et al., 2009; Šimo et al., 2017). These complex processes allow the ticks to remain attached for a prolonged period and compensate for different host defense mechanisms and requirements of the tick feeding cycle. Tick salivary molecules can be grouped into major categories based on their function, such as lipocalins (acting as kratagonists of biogenic amines and prostanoids), metalloproteases (having fibrinolytic activity), Kunitz-domain containing peptides (having putative sodium channel blocking or anticlotting activities), basic tail polypeptides (a unique protein family having anti-thrombin or plasminogen-activating activities), the salp-15 family (having immunosuppressive activity), the Isac family (having anti-complement activity), glycine-rich peptides (immunogenic components of the tick cement) (Francischetti et al., 2009; Blisnick et al., 2017; Chmelar et al., 2017; Šimo et al., 2017). Interestingly, not all molecules are secreted simultaneously (Kotsyfakis et al., 2015). This indicates that ticks adapt the composition of their sialomes (from the Greek, sialo = saliva) to the time of feeding, to their hosts, to the presence of pathogens, or to stress signals (Liu et al., 2014; Chmelar et al., 2016; Šimo et al., 2017; Tirloni et al., 2019). However, the mechanism of this adaptation is unknown.

In addition to creating a favorable microenvironment to acquire blood, the immunomodulatory functions of tick salivary molecules have been demonstrated to facilitate pathogen transmission. Tick saliva initially alters the inflammatory state of the inoculation site by altering the expression of cytokines (Thangamani et al., 2017) to increase neutrophil and monocyte (Hermance et al., 2016) influx. This results in an inflammatory environment that is conducive to viral invasion and macrophage-driven replication and dissemination (Labuda et al., 1996; Hermance et al., 2016). Elements of the tick saliva can also alter the behavior of dendritic cells to increase the replication of TBEV, in turn accelerating viral dissemination through the lymphatic system (Fialova et al., 2010; Lieskovska et al., 2018). Part of this effect appears to be due to lipocalins which target dendritic cells by binding to surface cholesterol (Roversi et al., 2017). Other affected cells include leukocytes (Rodriguez-Valle et al., 2013), natural killer cells, and other skin residents. Additional salivary factors can alter inflammation by targeting pro-inflammatory signaling molecules such as histamine, serotonin, leukotrienes, and complement (Francischetti et al., 2009; Šimo et al., 2017; Štibrániová et al., 2019).

The presence of tick saliva has been shown to increase both the acquisition and transmission of viruses. For example, ticks fed on animals inoculated with Thogoto virus have been shown to acquire infection at a higher rate when the inoculate also contained tick salivary gland extract (SGE) (Jones et al., 1989); the same has been observed with TBEV (Labuda et al., 1993). Experiments with Powassan virus (POWV), which is closely related to TBEV, had demonstrated that the amount of virus required to produce lethal animal infection was significantly lower when tick SGE was present (Hermance and Thangamani, 2015). This suggests that tick-borne viruses have evolved to exploit immunological events in the vertebrate hosts evoked by ticks to facilitate their feeding (Kazimírová et al., 2017). It is also plausible that the infection and replication of viruses in tick salivary glands induce alterations that enhance the transmission properties of tick saliva. For example, in contrast to uninfected ticks, the presence of POWV has been shown to increase skin inflammation during tick feeding (Hermance et al., 2016), which in turn increased the influx of macrophages and may potentially accelerate early viral infection and dissemination through the lymphatic system.

The potential of tick-borne viruses to alter the immunomodulatory properties of tick saliva to further enhance their own pathogenicity has not previously been accounted for in experiments involving the effect of tick saliva on viral transmission. This information is of great importance to understand the processes at the tick-host-virus interface and how these factors interact to produce early infection and systemic disease. In this manuscript, we describe for the first time the dynamic expression of tick salivary factors during the early stages of TBEV transmission and the implications of the components of the saliva of infected ticks in creating an immunomodulated feeding site.

## Materials and Methods

### Ticks and Animals

*Ixodes ricinus* ticks were obtained from a laboratory colony maintained at the Institute of Zoology, Slovak Academy of Sciences (Bratislava, Slovakia). BALB/c mice (females, 5-weeks-old) were purchased from Dobrá Voda Breeding Station (Institute of Experimental Pharmacology and Toxicology, Slovak Academy of Sciences). The mice were housed at the Institute of Virology (BMC SAS) under standard conditions. Food and water were provided *ad libitum*. Mice were 6 weeks old at the start of the experiments, and at the end of the experiments they were euthanized by cervical dislocation under anesthesia induced by carbon dioxide.

### Ethics Statement

The experiments involving laboratory mice were performed in accordance with the animal use protocol approved by the State Veterinary and Food Administration of the Slovak Republic (permit number 1335/12-221) and the Institute of Virology, Biomedical Research Center of the Slovak Academy of Sciences (BMC SAS).

### Tick Infection With TBEV, Feeding, and Dissection

F1 generation of laboratory-bred *I. ricinus* females were used for virus inoculation. TBEV (Hypr strain prepared as a 10% mouse brain suspension of 1.1 × 10^9^ PFU/ml in Leibovitz's L-15 medium) was provided by the Institute of Virology BMC SAS. Unfed *I. ricinus* females were inoculated with TBEV (5.5 × 10^4^ PFU per tick) through the coxal plate of the second pair of legs by a digital microinjector TM system (MINJ-D-CE; Tritech Research, Inc., USA) (for details see Thangamani et al., 2017) and subsequently kept at room temperature and 85% relative humidity in a desiccator for 21 days. The infection rate achieved by this procedure is ~100% (Slovák et al., 2014).

Two groups of BALB/c mice (*n* = 6 each) were infested with TBEV-infected or uninfected (control) *I. ricinus* females, respectively. The ticks were placed in small neoprene capsules glued on the shaved backs of the mice (two capsules per mouse, four tick females per capsule) (Thangamani et al., 2017). Ticks in each capsule were allowed to feed for either 1 or 3 h. After the allotted feeding time, mice (*n* = 3 per group and timepoint) were euthanized, and the attached ticks from one of the capsules were removed from the skin. The ticks and skin from the other capsules were used for the study by Thangamani et al. (2017). Ticks were dissected in chilled sterile PBS, pH 7.2, and their salivary glands were stored individually in either RNALater (uninfected ticks) or Tri Reagent (TBEV-infected ticks). Along with feeding ticks, salivary glands of unfed ticks (both TBEV-infected and uninfected) were prepared as described above. Samples in RNALater and TriReagent were stored at −80°C.

### Next Generation Sequencing

Three ticks (one tick per mouse) for each treatment condition (infected or non-infected) and each of the feeding timepoints were used in this study. RNA from the salivary glands of these ticks were extracted as described by us earlier (Hermance and Thangamani, 2015), and pooled prior to Illumina Next Generation Sequencing analysis. Illumina TruSeq v2 sample preparation kits were used for the RNA-Seq library construction. Each sample library was uniquely indexed to allow combining libraries during sequencing, and subsequent separation post-sequencing. Next generation sequencing (NGS) was performed at the UTMB NGS core facility. Sample libraries were analyzed by the Illumina HiSeq 1,500 using a 2 × 50 base paired end run protocol, with TruSeq v3 sequencing-by-synthesis chemistry.

### Bioinformatic Analysis

Bioinformatic analyses were conducted following the methods described previously (Chagas et al., 2013; Ribeiro et al., 2014), with some modifications. Briefly, the fastq files were trimmed of low-quality reads (<20), removed from contaminating primer sequences and concatenated for single-ended assembly using the Abyss (using k parameters from 25 to 95 in 10 fold increments) (Birol et al., 2009) and Trinity (Grabherr et al., 2011) assemblers. The combined fasta files were further assembled together with previous assemblies from *I. ricinus* (Schwarz et al., 2013; Kotsyfakis et al., 2015) using an iterative blast and CAP3 pipeline as previously described Karim et al. (2011). Coding sequences (CDS) were extracted based on the existence of a signal peptide in the longer open reading frame (ORF) and by similarities to other proteins found in the Refseq invertebrate database from the National Center for Biotechnology Information (NCBI), proteins from Acari deposited at NCBI's GenBank and from SwissProt. Reads for each library were mapped on the deducted CDS using the program blastn with a word size of 25, minimum identity of 97% and only one gap allowed. Functional classification of the transcripts was achieved by scanning the output of the different blast and rpsblast results using a vocabulary of ~400 words, the e value of the result and a result coverage > 75%. The classification of “unknown” was given if no informative match could be found. Read counts for each CDS were transformed to FPKM values. FPKM is a measure proportional to the relative molar abundance of each transcript.

Pairwise statistical comparisons between infected and uninfected libraries at the three different physiological states were assessed by a chi-squared test. The Bonferroni and FDR correction (Benjamini and Hochberg, 1995) were applied using the *P*-value package version 3.3.0 from the R software package (Team R. C., 2013). The normalized reads rate was determined by the expressions r1 × R2/[R1 × (r2 + 1)] e r2 × R1/[R2 × (r1 + 1)], in which r1 and r2 are the reads for each library (T– and T+) mapping to a particular transcript and R1 and R2 the number of total reads from each library mapped over all the CDS. One unit was added to the denominator to avoid division by zero. The Kruskall-Wallis statistical test (within the R package) was used to test for significant differences in FPKM values between transcripts within the different functional classes.

### Class Organization and Categorization

The list of identified transcripts was narrowed based on several parameters. Firstly, contigs with RPKM values of <10 were excluded. Transcripts were then selected based on having a p-value smaller than 0.05 for at least one timepoint (unfed ticks, 1 h post-attachment, and 3 h post attachment). The list was further narrowed to only proteins that were likely to be secreted as defined by the presence of secretion-related sequences or the predicted function of the protein being identified as salivary in nature (i.e., lipocalins or cystatins).

**Figure 1:**
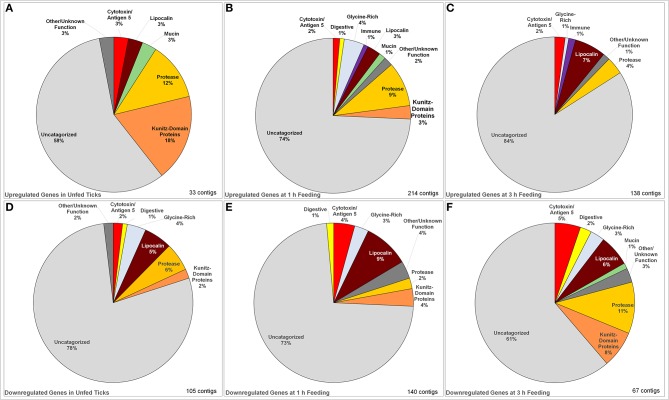
Identified categories of saliva-associated genes in *Ixodes ricinus* ticks upregulated and downregulated in response to tick infection with TBEV. Upregulation was observed in unfed ticks **(A)**, ticks after 1 h of feeding **(B)**, and ticks after 3 h of feeding **(C)** showing an overall increase in the number of genes upregulated. The dominant change in upregulation is in uncategorized genes, with the proportion of proteases and serine protease inhibitors decreasing with feeding while upregulation of lipocalins increase. Downregulation was also observed in unfed ticks **(D)** as well as those at 1 h **(E)** and 3 h **(F)** of feeding. In downregulated genes, the proportion of uncharacterized genes decreased while the downregulation of proteases, lipocalins, serine protease inhibitors, and cytotoxins became dominant.

These genes were then organized based on functional class, the results of which are presented in Figure 1, with a specific focus only on those components that are transferred to the host via saliva to modulate the host response to tick feeding (as opposed to metabolic or signaling genes within the salivary gland itself). Genes were identified as upregulated if expression in infected ticks was at least 10-fold higher than in uninfected ticks for at least one timepoint. Downregulated genes were defined as any that were downregulated below 0.1-fold during active tick feeding at 1 and 3-h timepoints as well as in cases where a gene was downregulated at any timepoint and upregulated at another. This relatively high 10-fold threshold was used to avoid false positives. This list was compiled into Tables 1, 2.

**Table 1:** Regulation of individual contigs and associated proteins in response to TBEV infection in salivary glands of unfed *Ixodes ricinus* ticks and at one and three hours of feeding organized by category, showing proteases and miscellaneous genes.

**Contig name**	**Miscallaneous**	**Unfed fold change**	**1 h fold change**	**3 h fold change**	**Specific type**	**Regulation pattern**
IrSigP-350116_FR2_1-200	scp gapr-1 like scp-like extracellular protein	0.59	0.00	0.00	Antigen 5	DOWN
IrSigP-120441_FR3_1-213	scp gapr-1 like scp-like extracellular protein	0.55	0.00	0.04	Antigen 6	DOWN
IrSigP-375532_FR3_1-137	U33-theraphotoxin-Cg1b	3.25	0.00	0.00	Cytotoxin	DOWN
IrSigP-257748_FR6_1-97	Cytotoxin-like protein	0.04	0.00	0.00	Cytotoxin	DOWN
Ir-343968	Cytotoxin-like protein	0.03	0.00	0.00	Cytotoxin	DOWN
IrSigP-228420_FR6_1-201	Antigen 5 protein	1.77	57.93	0.00	Antigen 5	UP
IrSigP-335607_FR3_98-231	Cytotoxin-like protein	3.54	18.25	0.00	Cytotoxin	UP
IrSigP-335605_FR1_100-362	Salivary secreted cytotoxin	2.47	22.11	0.01	Cytotoxin	UP
IrSigP-78898_FR3_6-103	Cytotoxin-like protein	0.20	0.39	23.10	Cytotoxin	UP
IrSigP-214219_FR3_66-354	Cytotoxin-like protein	0.37	0.04	19.57	Cytotoxin	UP
Ir-157968	Cytotoxin-like protein	0.17	0.23	19.25	Cytotoxin	UP
IrSigP-340386_FR3_43-459	Lipase	0.00	72.66	7.20	Lipase	UP
IrSigP-333694_FR2_33-438	Phospholipase	0.34	38.01	0.03	Lipase	UP
IrSigP-18398_FR2_13-108	5'-nucleotidase/apyrase	2.36	0.00	0.00	Nucleotidase	DOWN
Ir-389310	DDE superfamily endonuclease	1.03	0.06	0.00	Nucleotidase	DOWN
IrSigP-329586_FR4_1-305	Ficolin/ixoderin	1.54	11.74	0.05	Anticomplement	UP
IrSigP-270098_FR3_1-159	Microplusin preprotein	0.82	36.53	0.05	Antimicrobial	UP
IrSigP-72234_FR5_4-108	Defensin	1.77	0.31	27.60	Antimicrobial	UP
IrSigP-320485_FR1_6-333	Toll-like receptor 4	0.35	0.22	17.80	Unlnown	UP
IrSigP-229351_FR2_27-108	Salivary mucin	41.79	0.60	0.01	Mucous	UP
IrSigP-191860_FR2_160-436	Mucin-5ac	1.64	15.27	0.24	Mucous	UP
IrSigP-13558_FR4_1-129	Tick_mucins_17	0.43	16.99	0.03	Mucous	UP
IrSigP-355603_FR2_12-166	Tick_mucins_5	0.16	23.63	0.11	Mucous	UP
IrSigP-219752_FR5_71-232	Anticomplement protein ixac-b4 precusor	0.77	24.75	0.00	Anticomplement	UP
IrSigP-228937_FR5_31-448	Calreticulin	2.10	10.45	0.06	Calcium-Binding	UP
IrSigP-236871_FR5_46-121	5.3 kda protein	0.14	0.08	26.31	Antimicrobial	UP
Ir-230276	18.3_1–18.3 kda subfamily of the Basic tail superfamily	10.49	1.00	0.38	Unknown Function	UP
IrSigP-230278_FR2_12-166	18.3_1–18.3 kda subfamily of the Basic tail superfamily	0.02	264.75	0.46	Unknown Function	UP
IrSigP-4606_FR5_4-39-239	18.3_1–18.3 kda subfamily of the Basic tail superfamily	0.00	19.83	0.00	Unknown Function	UP
IrSigP-334686_FR1_85-367	23 kDa_1	0.51	0.03	32.41	Unknown Function	UP
IrSigP-384913_FR5_1-97	Secreted collagen-like peptide	1.18	0.00	0.00	Unknown Function	DOWN
IrSigP-344272_FR2_13-85	8.9 kda protein	3.08	0.00	0.00	Unknown Function	DOWN
IrSigP-5395_FR3_16-98	8.9 kda protein	1.19	0.00	0.00	Unknown Function	DOWN
**Contig name**	**Proteases**	**Unfed fold change**	**1 h fold change**	**3 h fold change**	**Specific type**	**Regulation pattern**
IrSigP-362100_FR2_36-293	Secreted metalloprotease	14.06	2.04	0.08	Metalloprotease	UP
IrSigP-256258_FR3_58-190	Secreted metalloprotease	61.09	12.59	0.00	Metalloprotease	UP
IrSigP-251827_FR3_33-213	Secreted metalloprotease	67.12	23.60	0.06	Metalloprotease	UP
IrSigP-362063_FR6_63-177	Dipeptidyl peptidase/kininase	16.11	4.64	0.00	Kininase	UP
Ir-364555	Secreted metalloprotease	0.00	10.24	0.00	Metalloprotease	UP
IrSigP-213551_FR6_1-495	Secreted metalloprotease	0.00	17.64	0.00	Metalloprotease	UP
IrSigP-230279_FR6_218-463	Secreted metalloprotease	0.41	18.78	0.64	Metalloprotease	UP
IrSigP-317111_FR6_39-280	Secreted metalloprotease	0.15	27.38	0.00	Metalloprotease	UP
IrSigP-213340_FR5_1-177	Secreted metalloprotease	2.05	27.28	0.08	Metalloprotease	UP
IrSigP-7165_FR5_1-150	Secreted metalloprotease	0.08	42.47	0.00	Metalloprotease	UP
IrSigP-213336_FR3_28-166	Secreted metalloprotease	3.03	42.20	0.00	Metalloprotease	UP
IrSigP-317110_FR2_1-150	Secreted metalloprotease	0.02	86.16	0.01	Metalloprotease	UP
IrSigP-218287_FR3_180-401	Secreted metalloprotease	0.00	110.78	0.43	Metalloprotease	UP
IrSigP-195061_FR1_23-118	Metalloprotease	0.90	11.21	0.01	Metalloprotease	UP
IrSigP-349107_FR2_284-526	Metalloprotease	1.69	14.64	0.10	Metalloprotease	UP
IrSigP-251870_FR1_13-811	M13 family peptidase	0.83	23.95	0.00	Neprilysin-Like Protease	UP
IrSigP-364331_FR3_106-370	Cathepsin B-like cysteine protease form 1	0.06	18.88	0.00	Cysteine Protease	UP
Ir-240260	Neutral endopeptidase-like protein	2.30	13.22	0.03	Neprilysin-Like Protease	UP
IrSigP-343497_FR6_11-110	Serin protease	0.30	10.81	0.00	Serine Protease	UP
IrSigP-371761_FR5_22-194	Typsin-like serin protease	1.18	32.41	0.15	Serine Protease	UP
IrSigP-305695_FR2_95-261	Typsin-like serin protease	1.79	67.28	2.19	Serine Protease	UP
IrSigP-365277_FR5_14-279	Typsin-like serin protease	1.90	11.94	0.03	Serine Protease	UP
IrSigP-305694_FR4_27-296	Typsin-like serin protease	1.69	49.62	0.64	Serine Protease	UP
IrSigP-371762_FR1_7-330	Typsin-like serin protease	1.61	102.54	0.45	Serine Protease	UP
IrSigP-299712_FR2_1-282	Secreted metalloprotease	0.45	0.95	18.61	Metalloprotease	UP
IrSigP-212742_FR1_38-533	Secreted metalloprotease	0.50	0.24	10.11	Metalloprotease	UP
Ir-263403	Peptidase family M28	0.35	0.03	11.55	Metalloprotease	UP
IrSigP-321314_FR5_72-813	M13 family peptidase	0.75	0.11	11.56	Neprilysin-Like Protease	UP
Ir-66057	Peptidase family m13	0.26	0.06	13.96	Neprilysin-Like Protease	UP
IrSigP-9195_FR5_47-292	Secreted metalloprotease	6.44	0.06	0.02	Metalloprotease	DOWN

*The dominant regulation pattern is determined to be “up” if at least one timepoint shows >10-fold increase or “down” if at least one timepoint shows a 10-fold decrease but no significant upregulation is observed*.

**Table 2:** Regulation of individual contigs and associated proteins in response to TBEV infection in salivary glands of unfed *Ixodes ricinus* ticks and at one and three hours of feeding organized by category, showing lipocalins, Kunitz-domain proteins, and glycine-rich family proteins.

**Contig name**	**Lipocalins**	**Unfed fold change**	**1 h fold change**	**3 h fold change**	**Regulation pattern**
IrSigP-221462_FR1_8-375	Salivary lipocalin	12.43	2.57	0.05	UP
IrSigP-220271_FR6_65-192	Lipocalin-1 1	0.00	20.17	0.00	UP
IrSigP-276153_FR6_14-222	Licpodalin-4_1 lipocalin	0.00	43.64	0.00	UP
IrSigP-183760_FR5_1-83	Lipocalin	0.64	16.63	0.76	UP
IrSigP-20975_FR4_21-174	Salivary lipocalin	0.00	39.02	0.00	UP
IrSigP-219616_FR4_1-149	Salivary lipocalin	10.14	14.18	0.00	UP
IrSigP-210096_FR5_1-211	Salivary lipocalin	0.00	78.32	0.43	UP
IrSigP-364550_FR2_70-334	Lipocal-1_37 lipocalin	1.41	0.04	47.83	UP
IrSigP-364548_FR2_70-353	Lipocal-1_37 lipocalin	2.01	0.00	17.35	UP
IrSigP-364544_FR2_70-350	Lipocal-1_37 lipocalin	0.14	0.09	13.01	UP
IrSigP-364543_FR5_1-246	Lipocal-1_37 lipocalin	0.30	0.45	10.52	UP
IrSigP-222396_FR3_1-106	Lipocalin-2 1	0.14	0.03	20.90	UP
IrSigP-288978_FR2_1-151	Lipocalin-2_1 lipocalin	0.37	0.00	78.29	UP
IrSigP-221393_FR4_1-262	Lipocalin-2_1 lipocalin	0.84	0.00	17.39	UP
IrSigP-318325_FR1_1-248	Lipocalin-2_1 lipocalin	1.05	0.07	13.28	UP
IrSigP-359698_FR2_22-253	Salivary lipocalin	0.17	0.14	111.28	UP
IrSigP-219614_FR6_1-112	Salivary lipocalin	8.69	0.00	0.00	DOWN
IrSigP-308104_FR5_9-217	Salivary lipocalin	5.61	0.00	0.00	DOWN
IrSigP-215089_FR1_281-606	Lipocalin	4.54	0.00	0.00	DOWN
IrSigP-333450_FR6_60-167	Salivary lipocalin	1.45	0.08	0.07	DOWN
IrSigP-231826_FR4_21-269	Lipocalin-2_1 Lipocalin	0.01	0.05	0.00	DOWN
IrSigP-232013_FR2_1-136	Lipocalin-2_1 lipocalin	0.00	0.00	0.00	DOWN
**Contig name**	**Kunitz-Domain proteins**	**Unfed fold change**	**1 h fold change**	**3 h fold change**	**Regulation pattern**
IrSigP-196594_FR3_14-286	BPTI/Kunitz family of serine protease inhibitors	16.33	2.64	0.03	UP
IrSigP-233783_FR4_27-108	Salivary kunitz domain protein	12.26	1.89	0.02	UP
IrSigP-194429_FR6_23-125	Salivary kunitz domain protein	16.16	0.24	0.00	UP
IrSigP-267369_FR6_1-92	Salivary kunitz domain protein	54.15	0.00	0.00	UP
IrSigP-247833_FR5_76-171	Tick kunitz 46	13.40	1.16	0.03	UP
IrSigP-138775_FR4_1-100	Tick_Kunitz_56	12.02	0.00	0.00	UP
IrSigP-226572_FR5_1-141	u3-aranetoxin-ce1a	10.98	0.28	0.00	UP
IrSigP-353563_FR5_1-97	Serine proteinase inhibitor (KU family)	0.00	61.68	0.00	UP
IrSigP-5685_FR5_27-338	Salivary kunitz domain protein	0.59	138.37	0.43	UP
IrSigP-300370_FR3_1-184	Tick kunitz 38	1.80	10.64	0.03	UP
IrSigP-286607_FR3_15-359	Tick_Kunitz_134	1.67	13.77	0.05	UP
IrSigP-389808_FR1_1-86	Tick_Kunitz_44	0.80	48.52	0.04	UP
IrSigP-362807_FR3_68-283	Tick_Kunitz_88	1.09	18.18	0.22	UP
IrSigP-292668_FR3_1-93	Tick_Kunitz_43	4.60	0.01	0.03	DOWN
IrSigP-249585_FR2_1-123	Tick_Kunitz_53	0.02	0.00	0.00	DOWN
IrSigP-257404_FR5_1-105	Salivary kunitz domain protein	2.49	0.03	0.01	DOWN
**Contig name**	**Glycine-Rick (Cement) proteins**	**Unfed fold change**	**1 h fold change**	**3 h fold change**	**Regulation pattern**
Ir-12879	GRP-2_441 Glycine rich family	0.00	14.03	0.00	UP
Ir-216707	GRP-2_441 Glycine rich family	2.28	19.63	0.02	UP
IrSigP-8569_FR6_25-160	GRP-2_441 Glycine rich family	2.26	46.52	0.02	UP
Ir-357614	GRP-2_441 Glycine rich family	3.54	63.88	0.00	UP
IrSigP-345293_FR5_35-172	GRP-2_441 Glycine rich family	0.00	83.52	0.46	UP
IrSigP-361126_FR6_1-133	GRP-2_441 Glycine rich family	1.80	105.26	0.00	UP
IrSigP-363306_FR5_53-179	GRP-2_441 Glycine rich family	0.18	151.07	0.47	UP
IrSigP-347965_FR2_15-168	GRP-2_441 Glycine rich family	0.02	168.29	0.11	UP
IrSigP-209173_FR2_40-179	GRP-2_449 Glycine rich family	0.00	20.34	0.64	UP
IrSigP-20709_FR3_56-211	GRP-2_590 Glycine rich family	0.49	0.05	23.37	UP
IrSigP-354603_FR4_25-133	GRP-2_471 Glycine rich family	1.91	0.00	0.00	DOWN

*The dominant regulation pattern is determined to be “up” if at least one timepoint shows >10-fold increase or “down” if at least one timepoint shows a 10-fold decrease but no significant upregulation is observed*.

Comparisons were derived from comparing expression changes of each functional class of predicted secreted salivary proteins at each timepoint.

### Evolutionary Relationships of *Ixodes ricinus* Lipocalins

The evolutionary history was inferred using the Neighbor-Joining method (Saitou and Nei, 1987). The bootstrap consensus tree inferred from 1000 replicates is taken to represent the evolutionary history of the taxa analyzed (Felsenstein, 1985). The percentage of replicate trees in which the associated taxa clustered together in the bootstrap test (1,000 replicates) are shown next to the branches. The evolutionary distances were computed using the Poisson correction method (Zuckerkandl et al., 1965) and are in the units of the number of amino acid substitutions per site. The analysis involved 106 amino acid sequences. All positions with <50% site coverage were eliminated. That is, fewer than 50% alignment gaps, missing data, and ambiguous bases were allowed at any position. There were a total of 196 positions in the final dataset. Evolutionary analyses were conducted in MEGA7 (Kumar et al., 2016). Clades with strong bootstrap support are numbered I–XXVII (Figure S2). Clades with red colored branches contain lipocalins detected in adult ticks feeding for 5 days (Beaufays et al., 2008a); these are marked with a red symbol. Branches of green color contain sequences that are upregulated at time zero when ticks are infected with TBEV; these are marked with a green square. Branches of turquoise color contain sequences that are upregulated at 1 h in TBEV-infected ticks; these are marked with a turquoise triangle.

### PCR Validation

Pooled tick RNA was converted into cDNA using a REPLI-g WTA Single Cell kit (Qiagen) in accordance with the kit instructions for purified total RNA. In brief, the kit generates cDNA from the RNA template, ligates the resulting cDNA into high molecular weight DNA molecules, and finally copies the ligated DNA using a proofreading DNA polymerase. This type of kit is more efficient than others and generates a larger quantity of cDNA for qPCR. The product of the kit was diluted 1:100 with molecular-grade water.

Targets of interest were identified based on the NGS data. The targets were required to be statistically significant at all three timepoints, to have an FPKM of ten or greater, and a change in expression of at least ten in at least one timepoint. Nineteen genes were selected from this group based on number of reads and the presence of sequence indicators representing secretion into the saliva.

Primers were designed based on sequencing data and ordered from Integrated DNA technologies. The sequences of these primers are listed in Table S1. They were reconstituted with molecular grade water and diluted to 10 μM. These samples were run in duplicate on a Biorad IQ5 machine using 10 μL Biorad iTaq Universal SYBR Green Mix, 0.6 μL of forward primer, 0.6 μL of reverse primer, 3.3 μL molecular grade water, and 2 μL diluted cDNA per sample. The thermal cycle included 50°C for 10 min, 95°C for 3 min, and then 45 cycles of 95°C for 15 s and 60°C for 30 s during which the samples were observed by the machine. After completion, melt curves were automatically generated, beginning with treatment at 95°C for 1 min followed by 55°C for 1 min and 81 observation cycles beginning at 55°C. The final results were baseline-normalized at an absorbance 100.

The Ct values between the technical duplicates were averaged, and changes in expression were calculated (Figure S1) using the ΔΔCt method, normalizing against the tick ribosomal S4 signal (Cabezas-Cruz et al., 2016).

## Results and Discussion

To understand the differential response of the *I. ricinus* salivary gland transcriptome during early stages of TBEV transmission, ticks were inoculated with TBEV by coxal microinjection 21 days prior to being fed on mice. This technique for infection is allowed for the generation of infected ticks with a high rate of success and has been widely accepted (Labuda et al., 2006; Slovák et al., 2014; Thangamani et al., 2017). Cohorts of infected and non-infected ticks were then allowed to feed on mice. At times 1 h and 3 h post attachment, ticks were removed from the mice for salivary gland dissection followed by RNA extraction. The RNAs were then sequenced and analyzed.

### Distribution of Changes in Gene Expression

Several classes of transcripts with significant salivary functions were identified as either up- or down-regulated in response to infection with TBEV. The numbers of genes with significant changes in regulation in each class in unfed, 1 h fed, and 3 h fed ticks are summarized in Table 3. In unfed ticks, only 33 genes were observed to be significantly upregulated in response to infection, as opposed to 214 after 1 h of tick attachment and 138 after 3 h. Comparing infected ticks to uninfected ticks, the number of downregulated genes in unfed infected ticks was 104, as opposed to 138 after 1 h of feeding and 174 after 3 h. This suggests that the strongest viral-induced changes to salivary expression occur after 1 h of feeding. This ability to modulate expression at a specific temporal point suggests that the virus has a direct and specific effect on gene regulation during the feeding process as opposed to a generic, global change in response to infection.

**Table 3:** Numbers of genes up and downegulated in response to TBEV infection in salivary glands of unfed *Ixodes ricinus* ticks and at one and three hours of feeding, categorized by predicted functional class.

**Class**	**Number of genes**					
	**Upregulated (Unfed)**	**Downregulated (Unfed)**	**Upregulated (1 h feeding)**	**Downregulated (1 h feeding)**	**Upregulated (3 h feeding)**	**Downregulated (3 h feeding)**
Cytotoxin/Antigen 5	1	2	3	6	3	9
Digestive	0	1	2	2	0	3
Glycine-Rich	0	4	9	2	1	6
Immune	0	0	2	0	2	2
Lipocalin	1	5	6	13	9	9
Mucin	1	0	3	0	0	2
Other/Unknown	1	2	4	5	2	6
Protease	4	6	20	3	5	18
Kunitz-Domain	6	2	6	5	0	13
Uncategorized	19	82	159	102	116	106
Total	33	104	214	138	138	174

The changes in expression skew heavily toward uncharacterized genes whose function is unknown (Figure 1), which make up between 58 and 84% of up- or down-regulated genes in each category. Proteases, serine protease inhibitors, and lipocalins tended to be the dominant functionally identifiable classes of genes, although their proportion of the total of up- or down-regulated genes varies substantially by timepoint. This was especially notable in unfed ticks, where 18% of the upregulated genes were identified as serpins or Kunitz-domain proteins, and 12% were proteases and metalloproteases. This proportion decreased during feeding so that after 3 h, lipocalins were more prevalently upregulated (7%) while serine protease inhibitors were not altered, and protease involvement had dropped to 4% of the genes. The opposite pattern is observed in downregulated genes: although a group of lipocalins was observed to be downregulated relatively consistently (5, 9, and 6% at unfed, 1 and 3 h timepoint, respectively), the proportion of downregulated proteases and serpin/Kunitz-domain proteins increased.

**Figure 2:**
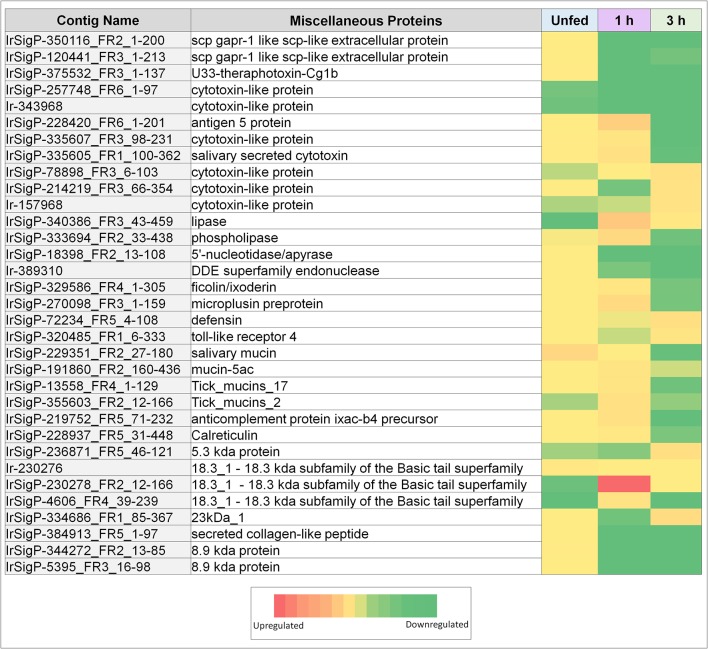
Heatmaps of several categories of genes identified in salivary glands of *Ixodes ricinus* ticks as up or down regulated in response to TBEV infection, including miscellaneous genes (cytotoxins, anti-microbial genes, and mucins). Regulation is generally most influenced at a single timepoint, often as the inverse of the pattern seen in other timepoints. Expression in each category is also not consistent between proteins, indicating that expression of each individual gene is not equivalent, and under complex regulatory control.

**Figure 3:**
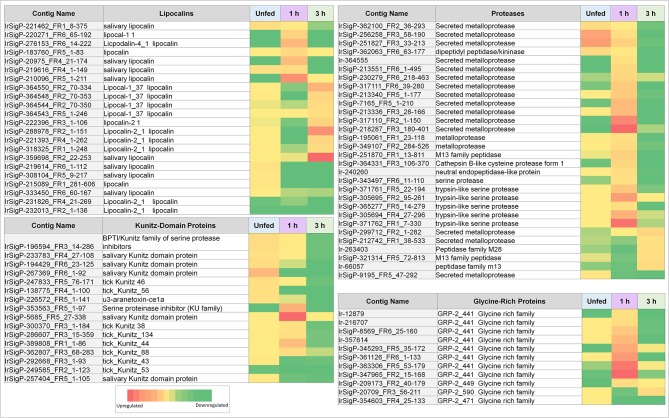
Heatmaps of several categories of genes identified in salivary glands of *Ixodes ricinus* ticks as up or down regulated in response to TBEV infection, including lipocalins, proteases, and protease inhibitors (largely Kunitz-type). Regulation is generally most influenced at a single timepoint, often as the inverse of the pattern seen in other timepoints. Expression in each category is also not consistent between proteins, indicating that expression of each individual gene is not equivalent and under complex regulatory control.

The expression of individual genes was also found to be remarkably inconstant across the three timepoints. None of the identified genes were found to be significantly upregulated at more than one timepoint. Many genes would often be upregulated at some points and downregulated at others (Tables 1, 2 and Figures 2, 3). These results indicate that viral interaction with the transcriptional regulation of salivary proteins is a highly dynamic process. Different sets of proteins are expressed at each individual timepoint in accordance with the stage of tick feeding; these, in turn, have different effects on the host immune response to both the tick and the virus it introduces.

## Implications Of Individual Protein Classes On Viral Behavior And Transmission

### Serine Protease Inhibitors

Serine protease inhibitors of the Kunitz family comprise a broad class of enzyme inhibitors that generally serve a regulatory purpose. In some animals, however, these inhibitors evolved to serve as venom (Mourao and Schwartz, 2013). For arachnids, Kunitz-domain-containing proteins either interfere with ion channel activity (Chen et al., 2012; Dai et al., 2012; Valdes and Moal, 2014; Santibañez-Lopez et al., 2018) or inhibit critical host proteins (Decrem et al., 2008; Bronsoms et al., 2011; Andreotti et al., 2012; Louw et al., 2013; Zhang et al., 2017). Tick saliva is essentially a form of venom evolved to facilitate blood-feeding, and although *Ixodes* spp. ticks retain some of their ion channel-manipulating Kunitz-domain peptides (Dai et al., 2012; Valdes and Moal, 2014), they largely fall within the enzyme inhibitor group.

Kunitz-domain serine protease inhibitors are known to inhibit coagulation (Dai et al., 2012; Chen et al., 2013; Louw et al., 2013; Assumpção et al., 2015; Zhang et al., 2017), angiogenesis (Drewes et al., 2012; Soares et al., 2016), and may reduce inflammation (Bronsoms et al., 2011). Their primary purpose is to ensure successful feeding by inhibiting blood coagulation. Multiple inhibitors are expressed to target several proteins involved in the coagulation cascade.

A steady supply of blood is required for a tick to continuously salivate. Unlike spiders, ticks have no equivalent to a venom sack and cannot synthesize and store substantial quantities of saliva in advance. It is instead produced in real-time during feeding, with the necessary fluid being provided as the bloodmeal is concentrated in the midgut. Viral transmission requires this continuous flow of outgoing fluid both as a vehicle for entry into the host and because the immunomodulatory portion of the saliva generates a microenvironment within the host that enhances viral infection (Thangamani et al., 2017).

As such, viral infection benefits from maintenance and enhancement of the anticoagulation system during the early stages of infection. Six Kunitz-type inhibitors and an arenetoxin analog (a probable trypsin inhibitor) were found to be upregulated in unfed ticks, with an average fold change of twenty and including one transcript that demonstrated a 54-fold increase. These represent a small amount of initial saliva prepared for an immediate and robust anticoagulant effect to facilitate more rapid early feeding. This group is not substantially upregulated at 1 h of feeding but shows downregulation when detected by 3 h with a fold change of 0.02–0.03.

Six additional Kunitz-type inhibitors were upregulated by infection after 1 h of feeding. The level of upregulation was more diverse at this timepoint than in unfed ticks, with three values having a fold change between 10 and 20, one having a change of 49-fold, one with a change of 62-fold, and one with a change of 138-fold. These contigs were not found upregulated at any other timepoint, and several were reduced at 3 h of feeding, including a change from 11 to 0.03 fold, 14 to 0.05 fold, and 49 to 0.04 fold.

At 3 h of feeding, the overall trend was a decreased expression of Kunitz-like inhibitors in response to viral infection, with no upregulated genes observed. Downregulation was not observed at any timepoint apart from 3 h in all but three genes, two of which were downregulated at 1 (0.008 and 0.03 fold) and one downregulated in uninfected ticks (0.02 fold). This downregulation continued for 3 h.

By 3 h, the ticks have already transmitted the infectious dose of the virus (Hermance and Thangamani, 2018) and have already imbibed a small supply of fluid from the host. Forced downregulation of the anticoagulant response interferes with feeding and may serve as a mechanism to trigger additional salivation (using already-consumed blood as a fluid source) as the tick attempts to compensate for increasingly poor fluidity of its bloodmeal. This process, in turn, forces the tick to inject more virus and immune-modulatory factors into the host. It may also lead to tick dislodgement of the host.

### Lipocalins

Lipocalins are proteins that bind to small organic molecules. Within the context of tick saliva, lipocalins are used to sequester histamine (Paesen et al., 1999; Sangamnatdej et al., 2002; Diaz-Martin et al., 2011; Valdes et al., 2016; Wang et al., 2016; Neelakanta et al., 2018), serotonin (Xu et al., 2013), leukotrienes (Beaufays et al., 2008a,b; Mans and Ribeiro, 2009), and other signaling molecules to reduce the anti-tick inflammatory response. A critical role of salivary lipocalins is to reduce itch and pain response resulting from histamine and serotonin signaling. This prevents from a scratching response of the host that could potentially damage the tick. Additionally, some salivary lipocalins have also often evolved secondary functions including anticoagulant activity (Mans and Ribeiro, 2008), as well as the ability to modulate the behavior of leukocytes (Rodriguez-Valle et al., 2013) and dendritic cells (Preston et al., 2013).

Our data show that lipocalins are differentially expressed at different timepoints during early phases of tick feeding, and the presence of TBEV causes both upregulation and downregulation of particular lipocalins. Unlike Kunitz-domain inhibitors, which tended toward early upregulation and later downregulation, lipocalins were more heavily upregulated at later timepoints but showed less significant changes in unfed ticks. Only two contigs showed upregulation in unfed, infected ticks with fold-changes of 12 and 10 compared to uninfected controls. By 1 h of feeding, six genes were upregulated in infected ticks vs. controls. This includes one of the genes that was upregulated in unfed ticks, which changed from 10- to a 14-fold increase. Other genes showed fold changes of 17, 20, 44, and 78, which represents a substantial change in several lipocalins.

At 3 h, nine genes were upregulated in infected ticks compared to uninfected ones. Six of these were between 10 and 21-fold. The remaining three showed fold changes of 48, 78, and 111. Although this trend is similar to what was observed at 1 h, the genes expressed are different at 3 h. Many of these 3-h genes were actually downregulated at 1 h, including 0.04 (48 at 3 h), 0.08 (13 at 3 h), 0.03 (21 at 3 h), 0.07 (13 at 3 h), and one as low as 0.0004 which became 17 at 3 h.

Additionally, six lipocalins were not found to be upregulated in infected ticks, but significantly downregulated compared to control ticks in at least one group.

Since *I. ricinus* lipocalins have been segregated into distinct phylogenetic groups (Beaufays et al., 2008a), we constructed a phylogram using the lipocalin sequences found in this study plus the lipocalin sequences described in Beaufays et al., (2008a), which were derived from transcripts of adult ticks feeding for 5 days. The resulting phylogram, made from 106 sequences, is complex, producing 27 clades with strong bootstrap support (Figure S2). Interestingly, the sequences that were upregulated at zero or 1 h are in different clades. All the sequences derived from 5 day feeding ticks inhabit clades that are not shared by those upregulated by TBEV. This is not surprising, since ticks switch their sialomes with time (Karim and Ribeiro, 2015; Perner et al., 2018), possibly as an immune evasion mechanism. Thus, the different clades may represent proteins with different functions, or with different antigenic character. Whether the different clades containing TBEV upregulated lipocalins at zero and 1 h represent different lipocalin functions or different antigenicity cannot be resolved.

From the perspective of viral infection, these results are complex. It has been previously demonstrated that the presence of TBEV causes an increased inflammatory reaction during a tick-bite (Thangamani et al., 2017). Viral transmission benefits from a pro-inflammatory response (Hermance and Thangamani, 2018) by summoning macrophages that can subsequently be infected and used to disseminate the virus to other tissues. By reducing histamine- and leukotriene-binding lipocalins, the host response to the saliva becomes more conducive to inflammation and consequently macrophage and neutrophil invasion. Additionally, modulating the behavior of dendritic cells and leukocytes by upregulated lipocalin expression has the overall effect of making the tissue less responsive to the presence of virus and thereby directing the brunt of the response against the tick. This altered expression increases the itch response, risking tick damage by host scratching. Since transmission of TBEV starts within a few minutes after tick attachment (Hermance and Thangamani, 2018), destruction of the tick does not outright prevent the transmission of virus, and in addition, pressure on the tick may in fact force the tick to partially regurgitate into the skin. Ticks that are unable to feed properly can dislodge themselves (Piesman, 1991) and attach to new hosts to attempt to complete the feeding, thus increasing virus transmission.

### Lectins

Lectins are carbohydrate-binding proteins found in tick saliva. Their exact role is poorly understood (Vechtova et al., 2018). Their function is thought to be immunomodulatory or antimicrobial (Sterba et al., 2011; Smith and Pal, 2014), although their agglutination property has also been linked to blood digestion in the midgut (Vechtova et al., 2018). It is possible that, as in the case of spiders, ticks begin some of their digestion process externally through saliva; in ticks, this would specifically occur within the blood-pool before ingestion. Ticks also actively modulate the lectin-mediated complement system, for example by the tick salivary lectin pathway inhibitor (TSLP1) (Hajdušek et al., 2013); the simultaneous production of ficolin-like lectins in tick saliva therefore appears counterintuitive unless it either fails to initiate the host complement cascade or acts as a direct competitor to host ficolin on lectin receptors.

Ixoderin, an analog of ficolin, was found to be upregulated in infected ticks at 1 h feeding (with a fold-change of 12) but downregulated compared to controls at the 3 h timepoint (fold-change 0.05). It is difficult to determine the exact function of this particular lectin within the context of TBEV infection, as it could either modulate the host response or be produced as a reaction to viral infection of the salivary gland. If the latter is true and the lectin targets glycosylated amino acids on the viral particles, it may serve as an adaptor to protect the virus from mammalian host response or to facilitate phagocytosis and infection of macrophages. Functional studies will be required to confirm if this viral/lectin interaction is common.

### Cystatins

Cystatins serve several functions within ticks, mainly with regard to hemoglobin digestion (Chmelar et al., 2017). Like tick lectins, they are present in tick saliva and also modulate host responses. Some inhibit TNF-α and IL12 production from dendritic cells and decrease CD4+ proliferation (Chmelar et al., 2017). TBEV replication in dendritic cells is enhanced by suppression of IFN signaling induced by cystatin exposure (Lieskovska et al., 2015). Cystatins have been shown to interfere with IL1β and IL18 in macrophages during *Anaplasma* infection and promote TBEV infection by further altering signaling in the dendritic cells. Cystatins bias the immune reaction away from the Th1 type that would be most effective against viral or intracellular pathogens (Schonenmeyer et al., 2017).

In our experiments, only one cystatin was observed with altered expression, although it is not listed in the presented tables and figures due to being down-regulated at only one of the required two exclusion points. It showed a fold-change of 0 (indicating that it was not detected in infected ticks) in unfed ticks and at 1 h, and then it showed a non-significant fold-change of 3 at 3 h.

This is unexpected, as the presence of cystatin is thought to enhance TBEV infection, yet is reduced here. The cystatin detected may be one of a more diverse group, may serve an alternate function and experience different regulation, as the sequencing data cannot positively identify it as cystatin 2, the salivary version. In this case, it probably serves a regulatory function in the way cystatin 3 does in human cells.

### Glycine-Rich Proteins

Upon attaching to a host, ticks begin to secrete a thick proteinaceous cement to better secure their mouthparts to the host skin. This cement is immunogenic, and though largely structural it has an impact on modulating host responses. Glycine-rich proteins are one of the cement's characteristic constituents (Hollmann et al., 2018).

The presence of glycine-rich proteins is critical to the transmission of TBEV (Labuda et al., 2006). Mice vaccinated against a particular glycine-rich protein proved resistant to TBEV transmitted by ticks. This vaccine was derived from a cement protein from *Rhipicephalus appendiculatus*, but proved cross-protective against proteins from *I. ricinus*, suggesting strong evolutionary conservation between the proteins as well as high homology of their respective immunogenic sites.

Our data show that most of the glycine-rich proteins identified were upregulated substantially at 1 h feeding in TBEV infected ticks. Of eleven genes detected, nine were upregulated. Three were found upregulated between 10 and 20 fold, three between 45 and 85 fold, and three higher than 100 fold. Only one gene was upregulated at 3 h with a fold change of 23, and the genes that were upregulated at 1 h were either not significantly changed or slightly downregulated. This most likely corresponds to a shift in the stage of cement deposition, with the viral-altered portion of the cement being applied early in the feeding and therefore of high early immunological relevance. These results imply that the production of cement has either increased or its composition has been altered in favor of its glycine-rich component. This corresponds with our observation that the production of tick mucin, another component of tick cement, was upregulated in response to infection at the 1-h timepoint. Of four mucin genes detected, three were upregulated 15, 17, and 24-fold, respectively in infected ticks compared to controls. The additional expression of glycine-rich proteins in the tick cement suggests a direct benefit to TBEV transmission.

### Proteases

The presence of proteases in tick saliva allows the tick to actively degrade host proteins that would otherwise impede its feeding activity. Ticks primarily employ reprolysin-like metalloproteases that directly degrade fibrinogen and the extracellular matrix (Francischetti et al., 2003; Mans et al., 2003; Harnoi et al., 2007; Barnard et al., 2012; Ali et al., 2014, 2015), though they also employ dipeptidyl carboxypeptidase to inactivate bradykinin (Ribeiro and Mather, 1998; Bastiani et al., 2002), a pain-inducing peptide. Metalloproteases are required for tick feeding; when animals are vaccinated against them, ticks feed poorly or die during the feeding process, usually in the later stages (post 24-h) (Decrem et al., 2008; Ali et al., 2015).

Similar to serine protease inhibitors, upregulation of metalloproteases increases the potency of the tick saliva's anticoagulant effect. Tick-borne viruses benefit when the tick consumes greater amounts of blood faster and is able to salivate more. Although these salivary compounds are usually associated with long-term (24 h or longer) tick feeding, the presence of TBEV causes their marked downregulation in infected ticks. These included a change of 61, 67, 14, and 16-fold, with the latter being the only definitive kininase detected. Of these four, those with the fold changes of 61 and 67 were still upregulated at 1 h of feeding, though to a lower extent (13 and 24-fold, respectively). Two genes were found downregulated (0.02 and 0.06-fold).

More infection-upregulated genes were identified at the 1-h timepoint. Of these 22 genes, fourteen had fold changes between 10 and 30, six had fold-changes between 30 and 90, and two had fold-changes of >100. Only three genes were found downregulated at this timepoint, with fold changes of 0.03, 0.06, and 0.05.

In contrast, nine protease contigs were found decreased in the presence of TBEV at the 3 h timepoint (0.01–0.08). Five were found upregulated, with four between 10 and 15-fold and one at 19-fold.

The expression of proteases mirrors our observations with serine protease inhibitors, where the presence of virus increases anticoagulant activity in the first two timepoints but downregulates it in the third. Likewise, this will cause the ticks to struggle to feed and force them to salivate more, thereby introducing more immunomodulatory compounds and virus as they attempt to continue feeding. They may also detach from the host without a blood meal, and may reattach to another, thus increasing virus transmission.

It should also be noted that while reprolysin-like or even astrolycin-like metalloproteases have been studied in ticks, these data have additionally identified several neprilysin-like proteases, a cysteine protease, and several serine proteases. The presence of serine proteases was largely upregulated at the 1 h timepoint and may correspond with the production of Kunitz-domain serine proteinase inhibitors. In this context the Kunitz proteins may serve to inhibit the serine proteases prior to saliva release to prevent damage to the tick salivary gland, with both assuming separate functions after release into the host.

### Other Tick Genes

Several miscellaneous genes of immunological and secretory importance have also been observed in this study. Calreticulin was found to be upregulated in infected ticks during the 1 h timepoint. In tick saliva, calreticulin has been shown to act as an immune signaling compound in addition to its normal calcium-binding function. It was found to contribute to the transmission of *Babesia* (Hajdušek et al., 2013) and may be of benefit to TBEV as well.

Several antimicrobial compounds also showed changes in expression. These include defensin, which was upregulated in infected ticks at the 3 h timepoint (fold change of 28), as well as a 5.3 kDa protein, which was also upregulated at 3 h (fold-change of 26) despite being slightly downregulated at 1 h feeding (fold-change 0.08). Microplusin was highly upregulated in infected ticks vs. controls (fold-change of 37), but then downregulated at 3 h (fold-change of 0.05), suggesting a decrease in tick response to the pathogen. Microplusin, however, is considered to be an antifungal and antibacterial compound (Silva et al., 2009) whereas defensin and possibly the 5.3 kDa protein can interact with viruses. Its role, therefore, may be part of a generic tick immune response to TBEV. These antimicrobial responses are probably not meant to interact with the host during feeding, but rather the result of the salivary glands reacting to their own infection. The temporal aspects are the result of changing gene expression during feeding.

Another category of molecules that is associated with cytotoxicity is represented by antigen-5. Antigen-5 is a compound of unknown function in insect venoms that results in potent allergenic response. Here, antigen-5 was found to be upregulated at the 1-h timepoint in infected ticks and then downregulated at 3 h feeding, resulting in a more powerful inflammatory response, as has been observed due to TBEV-infected ticks (Thangamani et al., 2017). This may serve to drive macrophages to the site of inoculation for infection and pathogen dissemination. Contributing to this effect are several cytotoxins found to be upregulated at 1 (3 genes) and 3 h (three genes) in infected ticks. These imply increased toxicity of the saliva, and the possibility of pro-inflammatory molecules being released from damaged or necrotic cells. Upregulation of this process may serve a direct benefit to the virus through increased inflammation. However, it will be necessary to perform deeper analysis of this gene and the structure and function of its product to fully understand what role this protein has in tick feeding, and what effect its upregulation may have on the transmission of TBEV.

One additional gene of interest was a member of the 18.3 kDa subfamily. The role of this type of protein in tick/host interaction is not fully understood and further specificities of this protein could not be identified immediately from the sequencing data. While its function is unknown, it showed the highest upregulation of any identified gene with a fold-change of 265 at the 1-h timepoint in infected ticks vs. uninfected ticks. It was downregulated in unfed infected ticks with a fold-change of 0.02, while its regulation was not significantly altered at the 3 h timepoint.

### Uncategorized Genes

A substantial number (>75%) of the genes recovered could not be identified. All of those listed possess signals that identify them as secreted compounds, and they all show differential expression at one or more timepoint. Their role cannot be ascertained at present.

## Conclusion

Tick saliva is essential to the tick's ability to modulate host hemostasis and inflammatory and immune responses against a tick during feeding and pathogen transmission. Our study clearly indicates a temporal variation in the tick salivary transcriptome expression during the first 3 h of feeding. Further, the pattern of expression is significantly impacted by infection of the tick with TBEV. The resulting changes to salivary transcriptome create an environment that is favorable to early viral infection and dissemination by altering compounds which affect the host immune system and hemostatic pathways. The early downregulation of lipocalins, metalloproteases and Kunitz-domain containing proteins by TBEV infection suggests that the tick will struggle to counteract host hemostatic and inflammatory responses and may be dislodged. Reattachment to another host would increase virus transmission. This strategy appears to be similar in effect to the method employed by *Yersinia pestis* where the bacillus impairs the ingestion of blood by infected fleas, leading them to bite multiple hosts in an attempt to feed (Hinnebusch et al., 2017). Tick saliva additionally contains compounds that facilitate tick infection, as determined by previously observed experimental evidence that tick saliva has pro-viral effect on infection; accordingly, this suggests that the virus has adapted in such a way as to not only take advantage of the immunomodulatory properties of the vector's saliva but to adjust the composition of the saliva in a way that benefits its own transmission. The exact mechanism of how the virus interacts with the vector's cellular machinery to alter transcription in this way is still unknown and it will be the subject of future investigations.

## Data Availability Statement

The raw reads used in this article were deposited at the Sequence Read Archive (SRA) of the National Center for Biotechnology Information (NCBI) under bioproject PRJNA576167, Biosample SAMN12956709, and runs SRR10240055, SRR10240054, SRR10240053, SRR10240052, SRR10240051, and SRR10240050. Seventy-three novel coding sequences have been deposited to DDBJ/EMBL/GenBank through the Transcriptome Shotgun Annotation portal under the accession GHXN00000000. The version described in this paper is the first version, GHXN01000000. The hyperlinked spreadsheet mapping the coding sequences, their annotation and statistical analysis can be downloaded from https://proj-bip-prod-publicread.s3.amazonaws.com/transcriptome/Ixric-TBEV-2019/Ir-tbev.zip.

## Ethics Statement

The experiments involving laboratory mice were performed in accordance with the animal use protocol approved by the State Veterinary and Food Administration of the Slovak Republic (permit number 1335/12-221) and the Institute of Virology, Biomedical Research Center of the Slovak Academy of Sciences (BMC SAS).

## Author Contributions

ST and MK designed the experiments, provided reagents, and materials and performed the experiments. JR, CH, and ST analyzed the data. CH and ST drafted the manuscript. All authors critically read and revised the manuscript.

### Conflict of Interest

The authors declare that the research was conducted in the absence of any commercial or financial relationships that could be construed as a potential conflict of interest. The reviewer JR declared a past co-authorship with one of the authors MK to the handling editor.

## Supplementary Material

Table S1 A list of primers utilized for PCR validation for this experiment.

Figure S1 Expression changes as determined by qPCR and the ΔΔCt method for ten genes showing a change in expression of >10 or <0.1-fold for at least one timepoint.

Figure S2 Evolutionary relationships of *Ixodes ricinus* lipocalins.
