# Supplementary material for: Tick-Borne Encephalitis Virus Infection Alters the Sialome of Ixodes ricinus Ticks During the Earliest Stages of Feeding
Source: Front Cell Infect Microbiol. 2020 Feb 18;10:41. doi: 10.3389/fcimb.2020.00041 (PMC7041427; doi:10.3389/fcimb.2020.00041)
Supplement: Table S1 — A list of primers utilized for PCR validation for this experiment. [file Table_1.pdf]

Contig Name	Target Name	Forward Sequence	Reverse Sequence
Ir-230276	18.3 kda subfamily of the Basic tail superfamily	5'-TgCACCACCgACTCTTATCA-3'	5'-ATTTTCgCAgTgTCCAAggT-3'
Ir-213731	Lipocalin 1_12	5'-TgTTATgTCAgCATCggAgC-3'	5'-gAAgACCTTggAAAgTgCgA-3'
IrSigP-247833_FR5_76-171	Tick Kunitz 46	5'-AgTCAgCTgCTTCTTCTCgg-3'	5'-CAACCAAACTCTgATTCgCA-3'
Ir-316614	Secreted metalloprotease fragment	5'-AAgCACAggAAgCAgACCAT-3'	5'-TCgAAAATTCACTATCCgCC-3'
IrSigP-334702_FR3_11-96	Tick salivary peptide group 1 fragment	5'-ACCAgTCCACCAACTATggg-3'	5'-ggTCTCATCgTTCCAgCAgT-3'
Ir-355603	Mucin	5'-TCgCACACgTAgTAggTggA-3'	5'-ACAAAATgCAACTgACggTg-3'
IrSigP-183760_FR5_1-83	Lipocalin	5'-gCggATATCAgAAggggATA-3'	5'-TggTAACAAACTTTCCAAgCg-3'
Ir-389808	Salivary Kunitz domain protein	5'-TgTTCTTgCCAgTTCCACAA-3'	5'-TCTgCAgTCgTgCTCATTTC-3'
Ir-322242	M13 Peptidase fragment	5'-ggggCAgACATgTCgTTTAT-3'	5'-CCTTATAggCTTCCACAgCg-3'
Ir-210553	M13 Peptidase fragment	5'-TCTACTCgTCTggACTgCCA-3'	5'-ggATTgAAATTCTgCgTCgT-3'
Ir-295490	Cytotoxin-like protein	5'-ggTgCCATgggTATCAgAAA-3'	5'-gCTgTAAACTgCCCTgAAgC-3'
Ir-211379<br>Ir-332301	Antigen 5	5'-CATACCgTgTgACCTgTTCg-3'	5'-TCATgTAgCACCTgCTCTCg-3'
Ir-211379	SCP GAPR-1-like protein	5'-CAAggACAgAAgCTTgggAg-3'	5'-AAggTAgTgggCCCAgTTTT-3'
Ir-332301	Antigen 5	5'-CATACCgTgTgACCTgTTCg-3'	5'-TCATgTAgCACCTgCTCTCg-3'
IrSigP-20709_FR3_56-211	Glycine-rich protein fragment	5'-TCCCgATCATTgCTgTTgTA-3'	5'-CCgTTTTTCCgTTCTTgAAA-3'
IrSigP-364544_FR2_70-350	Lipocalin 1_37	5'-ACCggAAAATgTgTCAgCTT-3'	5'-gATgTCgTCgACTgTgCTgT-3'
Ir-231127	Partial lipocalin	5'-AgCCAggTTgCAgCAATAAA-3'	5'-AggATCTTggAgTCCCgAgT-3'
IrSigP-318325_FR1_1-248	Lipocalin 2_1	5'-CCTgTACCTCgAgCATgTgA-3'	5'-TggggTAgCTgTTTCCTCTg-3'
IrSigP-214219_FR3_66-354	Cytotoxin-like protein	5'-CgATggCTCAAAATggAAgT-3'	5'-gTgCggTgCgTTAATTCTTT-3'
NA	Tick Ribosomal S4	5'-ggTgAAgAAgATTgTCAAgCAgAg-3'	5'-TgAAgCCAgCAgggTAgTTTg-3'
