# Supplementary figures and images for: Tick-Borne Encephalitis Virus Infection Alters the Sialome of Ixodes ricinus Ticks During the Earliest Stages of Feeding
Source: Front Cell Infect Microbiol. 2020 Feb 18;10:41. doi: 10.3389/fcimb.2020.00041 (PMC7041427; doi:10.3389/fcimb.2020.00041)

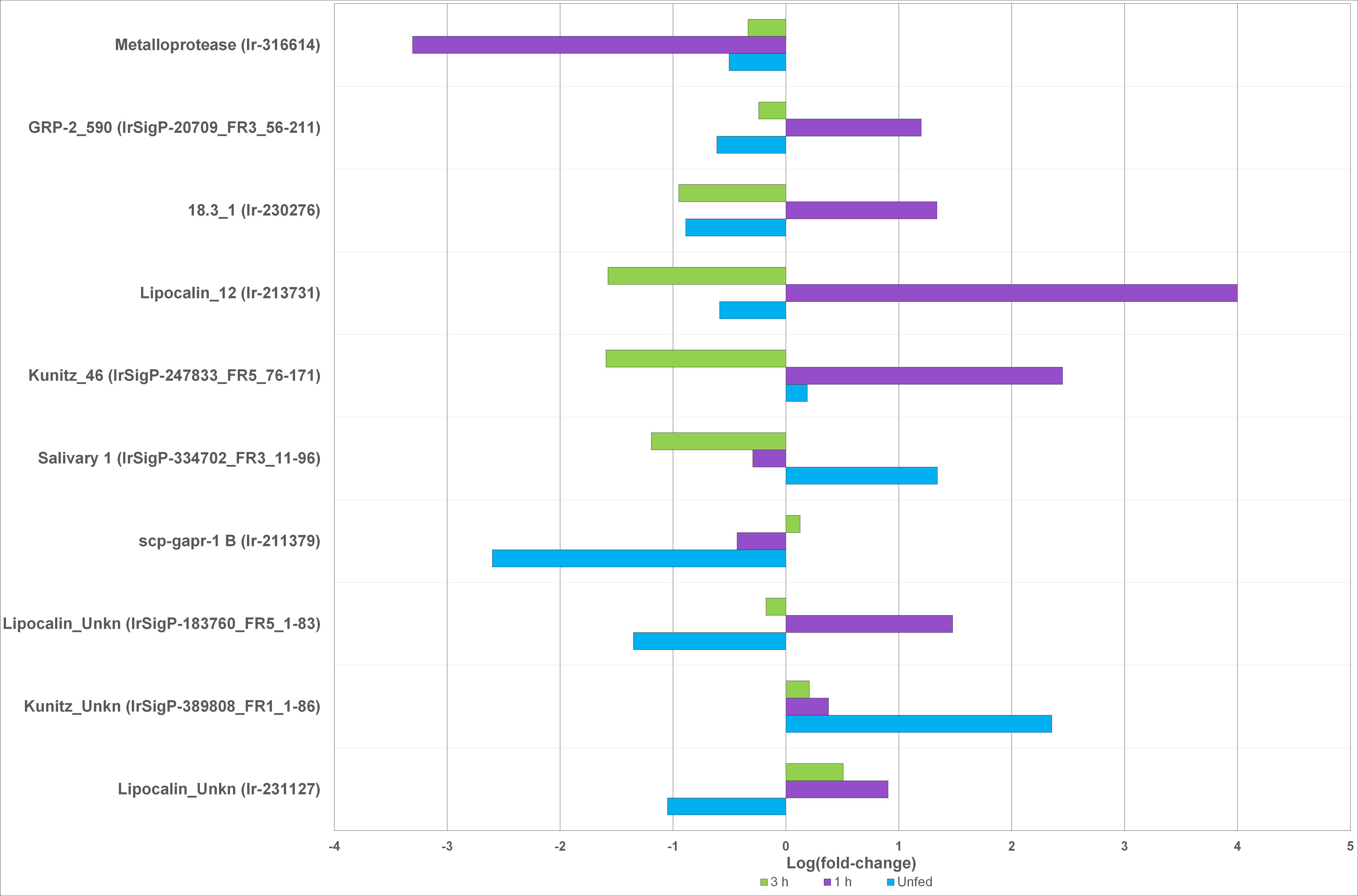

Supplement: Figure S1 — Expression changes as determined by qPCR and the ΔΔCt method for ten genes showing a change in expression of >10 or <0.1-fold for at least one timepoint. [file Image_1.tif]

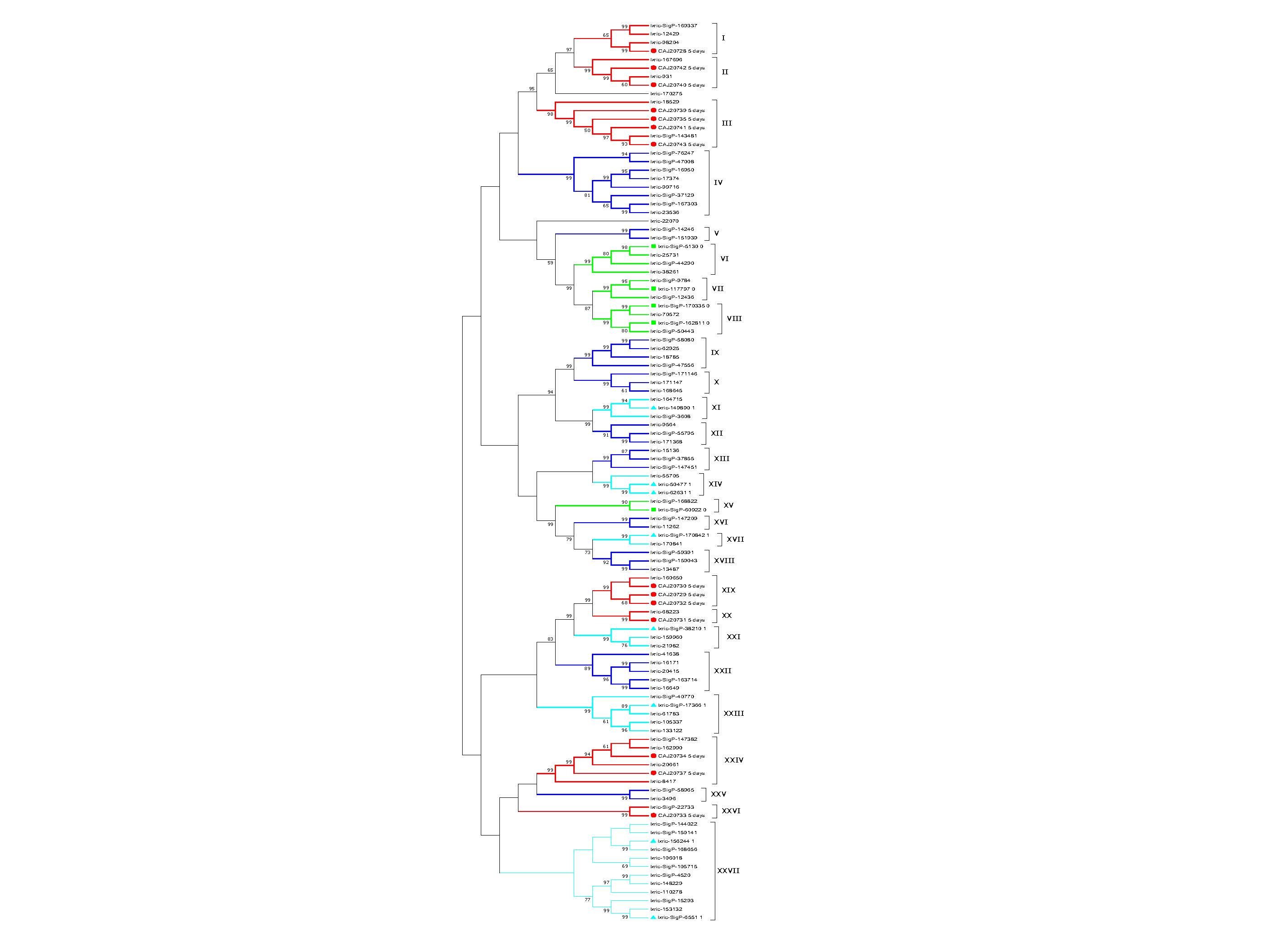

Supplement: Figure S2 — Evolutionary relationships of Ixodes ricinus lipocalins. [file Image_2.jpg]
